# Evaluation of Association between Blood Phenotypes A, B and AB and Feline Coronavirus Infection in Cats

**DOI:** 10.3390/pathogens11080917

**Published:** 2022-08-15

**Authors:** Eva Spada, Alice Carrera Nulla, Roberta Perego, Luciana Baggiani, Daniela Proverbio

**Affiliations:** Laboratorio di Ricerca di Medicina Emotrasfusionale Veterinaria (REVLab), Dipartimento di Medicina Veterinaria e Scienze Animali (DIVAS), Università degli Studi di Milano, 26900 Lodi, Italy

**Keywords:** feline coronavirus, cats, blood phenotypes, risk factors, epidemiology

## Abstract

Cats are susceptible to feline coronavirus (FCoV), a highly contagious virus with fecal–oral transmission. In people, susceptibility to coronavirus infection, such as SARS-CoV infection, has been associated with the ABO blood group, with individuals with blood group O having significantly lower risk of SARS-CoV infection. This study evaluated a possible association between feline blood group phenotypes A, B and AB and serostatus for antibodies against FCoV. We also investigated risk or protective factors associated with seropositivity for FCoV in the investigated population. Feline populations were surveyed for AB group system blood types and for presence of antibodies against FCoV. Blood phenotype, origin, breed, gender, reproductive status and age of cats were evaluated as protective or risk factors for coronavirus infection. No blood type was associated with FCoV seropositivity, for which being a colony stray cat (*p* = 0.0002, OR = 0.2, 95% CI: 0.14–0.54) or a domestic shorthair cat (*p* = 0.0075, OR = 0.2, 95% CI = 0.09–0.69) were protective factors. Based on results of this study, feline blood phenotypes A, B or AB do not seem to predispose cats to seropositivity for FCoV. Future studies on other feline blood types and other infections could clarify whether feline blood types could play a role in predisposing to, or protecting against, feline infections.

## 1. Introduction

Blood types are determined by antigens on the red blood cell (RBC) surface that can induce an immune response when transfused in individuals with different blood types. In people, many studies have shown that blood types can also play a role in susceptibility or resistance to infections caused by microorganisms, parasites and viruses [1,2,3,4,5,6,7]. Some blood types are in fact receptors for pathogens, and these can facilitate invasion of the cells or evasion of the host defense mechanisms [5,8,9,10,11].

In the last decade, many studies have found associations between human ABO blood types and susceptibility to coronavirus infection and reported in particular that blood type O individuals had a significantly lower risk of infection by severe acute respiratory syndrome coronavirus 2 (SARS-CoV-2) [12,13,14,15,16]. These findings can be explained if antibodies against blood type A and B have a protective activity against the transmission of ABO incompatible viruses. Blood type AB individuals lack both anti-A and anti-B antibodies; therefore, they would completely lack the protective effect given by these antibodies. In addition, the possibility of neutralizing the virus would be lower for type A and type B individuals than for type O individuals which have both anti-A and anti-B antibodies; thus, type A and B individuals could be expected to become more frequently infected than type O individuals [17].

While there is growing evidence in man that blood groups affect host susceptibility to infections [1,2,3,4,5,6,7,8,9], little is known about this in veterinary medicine [18,19]. To the authors’ knowledge, there is a single study reporting the role of blood group antigens in infection in non-human mammals. This is in an experimental study in rabbits in which histo-blood group antigens (HBGAs) were found to act as attachment factors for rabbit hemorrhagic disease virus (RHDV) with some HBGAs facilitating infection [18].

Cats have two blood group systems, the Mik system [20] and the major blood group system, the AB group system, which consists of three phenotypes, the most common type A, the rare blood type B and the extremely rare blood type AB [21,22,23,24]. Recently, five new feline blood types have been proposed [25]. The form of neuraminic acid on the RBC membrane is the major determinant of blood group antigens in the AB blood group system in cats [26,27]. N-acetyl-neuraminic acid (NeuNAc) is the unique disialoganglioside expressed on the membrane of type-B RBC, while type-A RBC have predominantly N-glycolyl-neuraminic acid (NeuNGc) containing gangliosides and limited quantities of NeuNAc gangliosides. Type-AB cats show equal amounts of NeuNAc and NeuNGc disialogangliosides on their RBCs [28,29]. In addition, blood type-A and B cats have natural preformed alloantibodies against blood type RBC antigens that they lack. Cats with blood type A have no, or limited quantity of, weak anti-B alloantibodies; type-B cats have high levels of strong anti-A alloantibodies, while blood type AB cats have no naturally occurring alloantibodies [22,30]. In feline medicine, knowing the blood type is important to prevent the risk of transfusion reaction due to blood type incompatibility and to prevent feline isoerythrolysis in type A kittens born from a blood type-B queen [31,32].

While the feline RBC AB blood group system antigens are biochemically unrelated to the human ABO antigens [21,26], there is a similarity between the human and feline blood group system: cats, like people, have naturally preformed alloantibodies against the blood type erythrocyte antigens they lack, with the exception of type AB cats which have no naturally occurring alloantibodies as they have both A and B antigens on the surface of their erythrocytes [30].

Cats are highly susceptible to feline coronavirus (FCoV), a highly contagious virus with fecal–oral transmission that is ubiquitous in multicat environments [33]. Antigenically, FCoV viruses can be classified as serotype I or II, with serotype I being the original and predominant type, while type II arises by recombination with canine coronaviruses [34,35]. FCoV frequently results in an asymptomatic infection, which can persist in some individuals. In some cats, in a sporadically and unpredictably way, the FCoV infection leads to feline infectious peritonitis (FIP), a highly fatal systemic infection [33]. The most commonly identified risk factors for FCoV infection are being a purebred cat and living in a multicat environment [33,36,37,38,39,40]. Evidence of the exposure to FCoV can be assessed identifying antibodies against the virus by using the gold standard FCoV indirect fluorescent antibody test (IFAT) or by indirect enzyme-linked immuno-sorbent assay (ELISA) [41,42].

Based on the knowledge of the association between human ABO blood types and susceptibility to SARS-CoV-2 infection, we performed this prospective evaluation on a possible association between feline blood phenotypes A, B and AB and serostatus for antibodies against FCoV. The second aim of this study was to investigate the risk, or protective role, of selected factors associated with seropositivity for this coronavirus in the evaluated population.

## 2. Results

In the sample population, 191/218 cats (87.6%) resulted of blood type A, 18/218 cats (8.3%) were type B and 9/218 cats (4.1%) were type AB. All type B and AB samples were confirmed by immunochromatographic method and by back-typing technique.

Seropositivity for FCoV was recorded in 84/218 samples (38.5%) and seronegativity in 134/218 (61.5%). No predisposing or protective role of blood type A, B or AB was found for FCoV seropositivity. In fact, FCoV seropositivity was recorded in cats of all blood phenotypes, with no statistically significant differences for any of the three blood phenotypes (Table 1). In addition, when antibody titers against FCoV were classified as high, medium, low and negative, there were no significant differences between the three blood phenotypes at Chi-squared test (*p* = 0.88441).

Results of univariate analysis (Chi-squared test) among factors other than blood phenotypes and seropositivity for FCoV antibodies are reported in Table 2. Factors of being a stray colony, owned, domestic shorthair (DSH), Persian, Ragdoll and Exotic Shorthair cat were significantly associated with seropositivity for FCoV.

Logistic regression analysis of significant seropositivity FCoV associated factors resulting from univariate analysis found only being a colony stray cat (*p* = 0.0002) and a DSH cat (*p* = 0.0075), but not an owned, Exotic Shorthair, Ragdoll and Persian cat to be independent factors significantly associated with FCoV seropositivity. OR for both colony stray and DSH cats were 0.2 (95% CI: 0.14–0.54 for stray colony cat, 0.09–0.69 for DSH cat). Therefore, being a colony stray cat and a DSH cat were protective factors with respect to FCoV seropositivity.

## 3. Discussion

In this study, we evaluated potential association and predisposing effects or protective role of feline AB blood group system phenotypes on FCoV infection based on the knowledge that ABO naturally occurring antibodies could influence the transmission of SARS-CoV-2 in man [17]. Association between blood type and infectious diseases in cats has not yet been demonstrated. A recent multicenter, multicountry study failed to demonstrate an association between feline AB blood groups phenotypes and genotypes and feline immunodeficiency virus (FIV) and feline leukemia virus (FeLV) infectious status [43]. The hypothesis for this study was that because the A blood type red cell antigen is also present on the lymphocytes of blood type A cats [44] and FIV and FeLV have feline lymphocytes as their target cells for pathogenicity [45,46], these viruses might interact with blood group lymphocyte antigens as receptors. Given the differential expression of blood group A on lymphocytes, the authors hypothesized that there would be a higher incidence of type A and AB in retrovirus-positive cats.

In our study, blood phenotypes showed no predisposition or protective effects against FCoV seropositivity. However, the origin of the cats investigated and, in particular, being a stray colony cat and being a certain breed such as a DSH cat, were protective factors against seropositivity. These results are not surprising as FCoV is a virus with fecal–oral transmission; therefore, the prevalence of FCoV infection and seropositivity is commonly associated with the density of cats housed together [33]. Stray colony cats, protected by law in Italy, are not confined like shelter and owned cats but are able to roam freely, frequently over large areas, can bury their feces outside and have close interaction with only a limited number of cats within the colony. This results in reduced FCoV fecal–oral transmission with respect to the situation in owned and shelter cats that frequently live in multicat environments where they share a litter box and food bowls with many other cats in the household [33,36,37]. In addition, management of stray colony cats is more in agreement with ethological and health needs of felines, resulting in reduced stress and stress-related infections.

The FCoV antibody seroprevalence of 38.5% in this study is similar to that reported in a previous study performed in Italy on 82 stray colony cats from Milan city in northern Italy [47], but lower than the prevalence of 81.6% in a population of 120 owned cats from southern Italy, most of which were from multicat environments as shelter or breeding cats [41]. Prevalence may be affected by the type of feline population investigated, since owned and, in particular, purebred breeding cats are more likely to be seropositive to FCoV, due to living in multicat environments and their intensive management that increase their exposure to FCoV infection and re-infection [33]. A previous finding that FCoV seroprevalence is higher in purebred cats compared to non-pedigree cats [37,38,39,40] was not apparent in our survey, probably because there were too few purebred cats to allow robust statistical comparisons. Moreover, even though the purebred cats in our population all belonged to different owners and did not come from a single or small number of breeding sites, we cannot exclude that some of the same breed cats could be related.

Our data confirmed that the prevalence of the three AB group system blood phenotypes in the populations evaluated in this study was similar to the feline blood group distributions reported by a previous study performed in the same area [22,23,48]. In all European feline breeds, in fact, the A blood type is the most common with 76% prevalence, followed by 21% of type-B and 3% of type-AB [24]. In particular, in cats from northern Italy, the prevalence of the three phenotypes has been reported to be the same as our population with 91% of type A cats, 5.2% of type B cats and 3.8% of type AB cats [22].

This study has a number of limitations. The FCoV serological test used in this study does not distinguish between type I FCoV infection and type II FCoV infection. Because the type I FCoV spike protein is different from the type II FCoV, the two serotypes of FCoV use different virus receptors, respectively. This means that the effect of blood type may differ by FCoV serotypes. In addition, the numbers of type B and AB cats are low in our population, and no attempt was made to increase the prevalence of these rare blood types. However, in our opinion this reflects the real interaction between feline blood types and the studied infection. Finally, we studied only the effect of blood phenotypes of the AB blood type system, and previously published evidence suggested that other, non-AB blood group systems, such as the Mik blood type, exist in cats [20]. Moreover, a more recent study [25] documented the presence of five distinct RBC antigens present outside the AB blood group system. In man, it is not only ABO blood group phenotypes that are associated with infectious disease, but many other blood group antigens are recognized as receptors for pathogens [9]. For example, Duffy blood group antigenic determinant Fy on the RBCs membrane affects susceptibility to *Plasmodium vivax* merozoites, the causal agent of malaria [11].

In conclusion, blood phenotypes A, B or AB seem to not predispose cats to seropositivity to FCoV. The recent identification of five novel feline erythrocyte antigens not belonging the AB blood group system in cats underlines how incomplete our knowledge on feline blood types is [25]. Future studies on these new blood types and others such as Mik blood type could clarify whether feline blood types other than AB blood group system could play a role in predisposing or protecting against feline infections.

## 4. Materials and Methods

This prospective observational study was performed between April 2021 and October 2021 in 218 cats from a number of provinces in northern Italy seen at the University of Milan and at the Ente Nazionale Protezione Animali (ENPA), section of Monza Brianza (MB). Feline patients for which residual blood samples (serum and whole blood in EDTA) from routine laboratory testing was available were enrolled in the study. The blood was typed and tested for anti-FCoV antibodies. In stray colony and shelter cats, blood samples, collected in EDTA and in plain tubes for serum samples, were specifically drawn for the study. Samples were taken from 218 cats; 81 were stray colony cats; 60 were shelter cats, and 77 were owned cats. Of these, 194 were DSH, and 23 were purebred cats. Most common breeds were seven Ragdolls, three Persians and three Exotic shorthair cats. For one cat, information regarding breed was missing. Of the cohort, 102 cats were males, 114 were female, and 36% of the cats were neutered. For two cats, information regarding sex was missing. Exact age was recorded for 201 cats and varied from 3 months to 20 years, with a median age of 2 years (25–75% percentiles: 1–6 years). A total of 110 cats were categorized as young (from birth to 2 years), 73 cats as adult (from 3 to 10 years) and 24 as senior (more than 11 years) [49].

### 4.1. FCoV Serological Testing

The FCoV antibody test kit Immunocomb^®^ (Biogal Galed Laboratories, Gibbutz Galed, Israel), an ELISA based serological technique, was used to test for the presence of antibodies to FCoV following the manufacturer’s instructions and as previously described [38] and validated [42,50]. The sensitivity and specificity of this FCoV test is 100% and 100%, respectively [42]. This test provides a semi-quantitative measure of the FCoV antibody present in whole blood, plasma or serum sample. The test produces grey spots, which are read on a scale from S1 to S6, depending on the intensity of the color of the spots. The interpretation of test results were: results equal to or greater than S3 were considered positive, readings of 2.5 or less were considered negative [50]. In addition, S3 and S4 was classified as low positive, S5 as medium positive and S6 as high positive [38,50].

### 4.2. Blood Typing and Back-Typing

Blood typing was performed at the Veterinary Transfusion Research Laboratory (REVLab), Department of Veterinary Medicine (DIMEVET), University of Milan, Italy using the gold standard agglutination on tube technique as previously described [2]. All samples determined to be type-B and AB with agglutination on tube technique were confirmed by an alternative blood typing method (an immunochromatographic test, LabTEST A+B, Alvedia, Limonest, France) and by the back-typing technique performed as previously described [51,52].

### 4.3. Statistical Analysis

Descriptive statistics were used to summarize demographics, FCoV serological status and blood phenotypes. Distribution of data was assessed using the d’Agostino-Pearson test. Associations between the three blood phenotypes and FCoV serological status were investigated by Chi-squared (χ^2^) test. Logistic regression using a stepwise method was used to test factors associated with seropositivity from univariate analysis. Hosmer–Lemeshow goodness of fit test was used to test for the model diagnostics. Significant results were considered for *p* < 0.05. Odds Ratio (OR) with 95% CI were calculated for factors with a significant association identified. Statistical analyses were conducted using commercially available software (MedCalc^®®^ Statistical Software version 20.112, MedCalc Software Ltd., cityOstend, Belgium).

## Figures and Tables

**Table 1:** Effect of feline blood phenotypes A, B and AB on serostatus for Feline coronavirus (FCoV) antibodies in an Italian population of 218 cats.

Blood Phenotype	FCoV		*p*–Value (Chi-squared Test)
	Seropositive n = 84 n (%)	Seronegative n = 134 n (%)	
Type A	75 (89.3%)	116 (86.5%)	0.5541
Type B	6 (7.1%)	12 (9.0%)	0.6369
Type AB	3 (3.6%)	6 (4.5%)	0.7440

**Table 2:** Characteristics of an Italian population of 218 cats investigated for antibodies against Feline coronavirus (FCoV) and analyzed for factors associated with seropositivity.

Parameter	Variable	FCoV		*p*–Value (Chi-squared test)
		Seropositive n = 84n (%)	Seronegative n = 134n (%)	
Origin n = 218	Stray colony cats	16 (19.0%)	65 (48.5%)	**<0.0001**
	Shelter cats	25 (29.8%)	35 (26.1%)	0.5588
	Owned cats	43 (51.2%)	34 (25.4%)	**0.0001**
Breed n = 217	DSH	67 (79.8%)	127 (94.8%)	**0.0003**
	British Shorthair	0 (0.0%)	2 (1.5%)	0.2617
	Maine Coon	0 (0.0%)	1 (0.7%)	0.4285
	Persian	3 (3.6%)	0 (0.0%)	**0.0280**
	Ragdoll	7 (8.3%)	0 (0.0%)	**0.0007**
	Scottish Fold	1 (1.2%)	1 (0.7%)	0.7384
	Siberian	2 (2.4%)	0 (0.0%)	0.0734
	Siamese	0 (0.0%)	2 (1.5%)	0.2617
	Chartreux	1 (1.2%)	0 (0.0%)	0.2066
	Exotic Shorthair	3 (3.6%)	0 (0.0%)	**0.0280**
Gender n = 216	Male	38 (45.2%)	64 (47.8%)	0.6420
	Female	46 (54.8%)	68 (50.7%)	
Reproductive Status n = 216	Neutered	35 (41.7%)	43 (32.1%)	0.1761
	Intact	49 (58.3%)	89 (66.4%)	
Age class n = 207	Young (0–2 yrs)	42 (50.0%)	68 (50.7%)	0.6546
	Adult (3–10 yrs)	31 (36.9%)	42 (31.3%)	0.5367
	Senior (>11 yrs)	9 (10.7%)	15 (11.2%)	0.8223

DSH: domestic shorthair; yrs: years; In bold, statistically significant value with *p* < 0.05 at univariate analysis (Chi-squared test).

## Data Availability

The original contributions presented in the study are included in the article, further inquiries can be directed to the corresponding authors.
